# *SeedExtractor*: An Open-Source GUI for Seed Image Analysis

**DOI:** 10.3389/fpls.2020.581546

**Published:** 2021-02-01

**Authors:** Feiyu Zhu, Puneet Paul, Waseem Hussain, Kyle Wallman, Balpreet K. Dhatt, Jaspreet Sandhu, Larissa Irvin, Gota Morota, Hongfeng Yu, Harkamal Walia

**Affiliations:** ^1^Department of Computer Science and Engineering, University of Nebraska-Lincoln, Lincoln, NE, United States; ^2^Department of Agronomy and Horticulture, University of Nebraska-Lincoln, Lincoln, NE, United States; ^3^Department of Animal and Poultry Sciences, Virginia Polytechnic Institute and State University, Blacksburg, VA, United States

**Keywords:** rice, image analysis, seed size, seed color, GWAS, genome wide analysis

## Abstract

Accurate measurement of seed size parameters is essential for both breeding efforts aimed at enhancing yields and basic research focused on discovering genetic components that regulate seed size. To address this need, we have developed an open-source graphical user interface (GUI) software, *SeedExtractor* that determines seed size and shape (including area, perimeter, length, width, circularity, and centroid), and seed color with capability to process a large number of images in a time-efficient manner. In this context, our application takes ∼2 s for analyzing an image, i.e., significantly less compared to the other tools. As this software is open-source, it can be modified by users to serve more specific needs. The adaptability of *SeedExtractor* was demonstrated by analyzing scanned seeds from multiple crops. We further validated the utility of this application by analyzing mature-rice seeds from 231 accessions in Rice Diversity Panel 1. The derived seed-size traits, such as seed length, width, were used for genome-wide association analysis. We identified known loci for regulating seed length (*GS3*) and width (*qSW5/GW5*) in rice, which demonstrates the accuracy of this application to extract seed phenotypes and accelerate trait discovery. In summary, we present a publicly available application that can be used to determine key yield-related traits in crops.

## Introduction

Most of the plant-based food that we eat is either seed or seed-derived products. Thus, a large proportion of resources in crop improvement programs are invested toward better seeds. In this context, obtaining precise measurements of seed size and seed shape is critical to both breeding programs aimed at enhancing crop yields and facilitating fundamental research that is focused on discovering genetic components that regulate seed size. Manual measurements of seed size provide evidence of restricted parameters such as length and width at a low resolution, which can be error-prone and time-consuming. Mechanized seed size measuring equipment is expensive, requires regular calibration, and often needs large amounts of seeds to run through the system. In contrast, imaging-based automated platforms that are tailored to accurately measure seed parameters offer an efficient solution to mitigate time constraints, seed amount issues, and circumvent manual errors (Furbank and Tester, 2011; Fiorani and Schurr, 2013; Sandhu et al., 2019; Yang et al., 2020). Moreover, high-throughput image analysis provides a powerful tool for trait discovery that facilities a more rapid input into downstream analysis such as genome-wide association studies (GWAS) for performing genetic mapping of yield-related traits.

Qualitative assessment of the yield-related traits can also be important to ensure optimal nutritional values of seeds (Zhao et al., 2020). Within this framework, seed color can be associated with enhanced nutrition (Shao et al., 2011 and references therein). For instance, colored rice varieties carry antioxidant properties, which are known to decrease the risks involved with developing cardiovascular diseases (Ling et al., 2001). Similarly, pigmented maize seeds offer several beneficial effects on human health due to their antioxidant properties (Casas et al., 2014; Petroni et al., 2014). In addition to their medicinal properties, colored rice varieties hold cultural significance for certain regions and are consequentially valued in the respective local markets (Finocchiaro et al., 2007). Furthermore, the red pigmented wheat, which is resistant to pre-harvest sprouting, has been extensively targeted in wheat breeding programs (Groos et al., 2002).

Keeping in view the importance of seed size and color, several seed image analysis applications have been developed. For example, *SmartGrain* determines seed morphometrics such as area, perimeter, length, and width, as well as seed shape. However, it does not extract seed color information (Tanabata et al., 2012). On the other hand, *GrainScan* provides information with respect to seed size and color (Whan et al., 2014). Both the applications can be operated only on the Windows platform. Although, these applications offer high levels of accuracy for analyzing seed images for size and shape determination, the adjustments that may be needed in setting the parameters are limited. For instance, *SmartGrain* only allows the user to determine the foreground and background colors, wherein *GrainScan* can only allow the user to set the size parameters. Moreover, processing a large number of images is time-consuming, and images with uneven illumination pose a challenge for precise measurements that may interfere with downstream analysis. These applications are not open-source and, therefore, cannot be further developed to improve based on user needs. In addition, other tools such as *SeedSize* (Moore et al., 2013) and Plant Computer Vision or Plant CV can be utilized to determine seed morphometrics (Fahlgren et al., 2015; Gehan et al., 2017).

To address the missing features in available seed image analysis software, we have developed a MATLAB based tool –*SeedExtractor*, an open-source graphical user interface (GUI) software that allows a user to conduct seed size analysis with precision. Based on the image processing libraries in MATLAB, our application is highly efficient, as it can process a large number of samples in a short period of time. The application allows the user to fine-tune the parameters for image processing and can handle a wide array of images. Most importantly, our application is open-source as the source code of our program is published and MATLAB is available to most users through institutional license. Moreover, we developed a Standalone version of *SeedExtractor*, which uses MATLAB Compiler Runtime and does not require MATLAB license for its operation. Overall, our tool allows the user to freely modify the application to suit more specific needs. As a test case to examine the value of this software, we screened mature seeds from 231 rice accessions corresponding to Rice diversity Panel 1 (RDP1) with different genetic (*indica, temperate japonica, tropical japonica, aus*, and *admixed*) and geographical backgrounds using*SeedExtractor*. The derived seed-size related traits such as mature seed length and width were used to perform GWAS. Our association mapping confirmed the identity of known loci/genes regulating seed length (*GS3*) and width (*qSW5*) in rice, thus validating the accuracy of this application tofacilitate genetic analysis and trait discovery.

## Materials and Methods

### *SeedExtractor* Workflow

**FIGURE 1:**
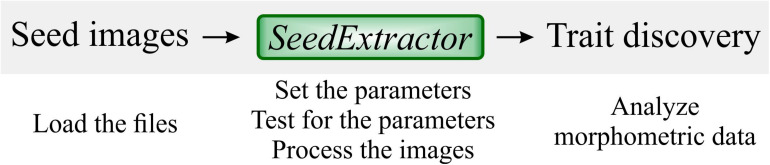
*SeedExtractor* workflow. Firstly, seed images are loaded, and the parameters are set. Testing of the parameters is performed to ensure optimal settings. Then, batch processing can be conducted to extract seed traits.

*SeedExtractor* is a MATLAB-based application, which makes it compatible with multiple operating systems. The tool is available in two formats: Standalone and Regular (see “Software Availability” section). The standalone version of *SeedExtractor* uses “MATLAB Compiler Runtime” and does not require MATLAB license for its operation. The regular version does require MATLAB license for its operation. Both these versions are similar in their interface and performance. First, the user needs to install the *SeedExtractor* application. Then, the folders which contain the seed images (scanned or camera-based images) must be provided (see Figure 1). Next, the parameters, based on user’s requirement, is set and an individual image is tested to validate the optimal settings (see Figure 1). Sequentially, batch processing can be conducted to extract seed traits such as (1) area, (2) perimeter, (3) major axis length (length), (4) minor axis length (width), (5) circularity, (7) seed number, (8) color intensity (different channels) and other digitally derived traits such as centroid. We have provided a step-by-step guide to use *SeedExtractor* (see *SeedExtractor* Guide Document: Supplementary Data Sheet 1).

### Software Implementation

#### Tool Development

**FIGURE 2:**
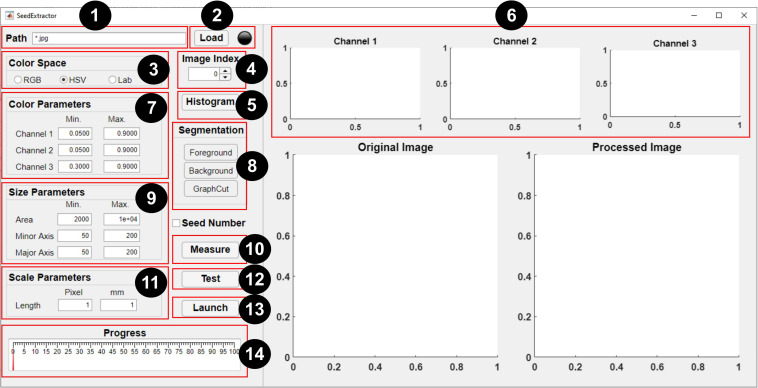
Graphical user interface of *SeedExtractor*. The numbers denote a step-by-step guide on how to use the application: (1) path of the seed images is specified (* represents that all images in the particular folder need evaluation), (2) files are loaded automatically, (3) selection of color space should be made, (4) spinner can be used to change the current image (shown in the original image), (5) the user may select “*histogram*” option (if applicable), (6) histograms representing distribution of colors in the three channels of the selected color space will be generated, (7) the range of histograms can be used to set the color parameters for the respective channel, (8) by selecting “*foreground*” and “*background*”—the user can scribble to define the color of the seed and background, respectively, and “*graph cut*” will facilitate segmentation of the seeds from the background, (9) minimum and maximum seed size parameters are defined (either default settings or manual corrections can be made) to filter out regions that are not seeds, (10) the user can “*measure*” objects that have been used as a scale in the image and (11) define the scale measurement (in millimeters) that will aid in transforming the pixel length into metric units, (12) a test run should be performed prior to batch processing in order to ensure that the parameter settings are optimized, (13) if the user has decided which parameters will be optimum, batch processing can be initiated, and (14) progress can be monitored via the progress bar.

We have designed a GUI based on MATLAB, which provides users with the flexibility of setting unique parameters for processing seed images (see Figure 2).

#### Execution Steps

A brief step-by-step guide is provided below to perform seed image analysis: (1) path specification, (2) file loading, (3) color space selection, (4) image selection, (5) histogram generation, (6) parameters setting, (7) graph cutting, (8) scale measurement, and (9) testing and processing.

##### Path Specification

*SeedExtractor* is compatible with widely used image formats including *jpg*, *png*, and *tiff*. This tool supports batch processing by loading all the images using a regular expression. For example,“FOLDER NAME*\^∗^.jpg*”loads all the *jpg* images under the respective folder.

##### File Loading

Once the correct regular expression has been typed in “*Path*” textbox, the “*Load*” button can be clicked to load all the filenames into the application. The “*Light bulb*” located on the right side of the interface will turn red while the filenames are being loaded. Afterward, the unprocessed image will be shown in “*Original Image*” (see Figure 2). The spinner can be used to change the index of the current image. The current image will be used for parameter setting and testing in later steps.

For accurate measurements, the “*Original Image*” and “*Processed Image*”can be zoomed in and out to check for any discrepancy between the original image and the processed image in the binary format. They can also be panned by holding the left-click button.

##### Color Space Selection

The application supports three different color spaces: (1) red, green, and blue (*RGB*),(2) hue, saturation, and value (*HSV*), and (3) *Lab*. These three different choices of color spaces provide the flexibility to the user in finding the optimal segmentation output. Once the color space is selected, the images will be processed in the respective color space for the next steps.

##### Histogram Generation

The three histograms (*Channel 1*, *2*, and *3*; see Figure 2) showing the distribution of colors in the three channels of the selected image (seed and the background) are generated. The meaning of the channels is dependent on the color space selected by the user. For example, if “*RGB*” is chosen as a preferred color space, then histograms for “*Channel 1*, *2*, and *3*” refer to “*red*, *green*, and *blue.*” Similarly, “*hue*, *saturation*, and *value*” for “*HSV*,” and “*l*, *a*, and *b*” for “*Lab*” color space. The distribution of colors in these three channels can be used as guide for setting the correct color ranges. Default parameters are provided; however, the user needs to change the color parameters in order to use their own preferred range of color channels based on the histograms (see *Graph Cutting*).

##### Parameter Setting

A set of default parameters are automatically loaded after launching the tool. Channel ranges (minimum and maximum) are used to segment the seed regions from the background. Minimum and maximum seed size and shape parameters, such as area, major and minor axis length, are used to filter out regions that are not seeds. However, the default parameters may not work for all the seed types or images. Thus, in this case, the user may need to set these parameters manually.

##### Graph Cutting

To simplify the process of parameter setting, our application can also generate the parameters automatically based on “*user scribbles*” to select the foreground and background. Then, using the “*GraphCut*” algorithm (Kwatra et al., 2003), the foreground can be segmented from the background.

**FIGURE 3:**
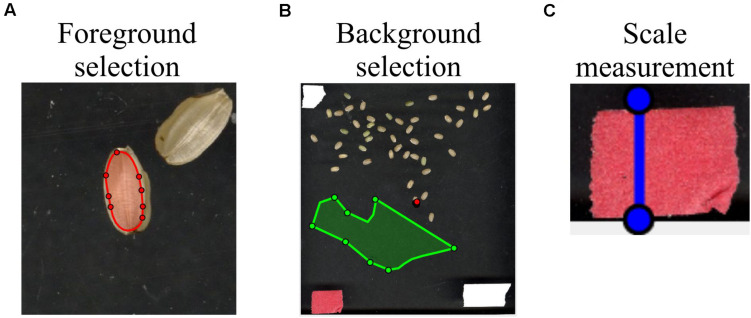
Selection of foreground, background, and scale measurements. By utilizing the function “*user scribbles*,” *SeedExtractor* can select foreground and background. **(A)** To select the foreground, the user can click the “*foreground*” button on the graphical user interface and scribble on the seed with a red mark. The image can be zoomed inward for the purpose of scribbling on smaller seeds. **(B)** For background selection, the user can click the “*background*” button and scribble on the background with a green mark. **(C)** For metric-scale measurements, the application allows the user to measure objects that have been used as a scale in the image, which can then be used to transform the pixel length into millimeters. For this, a blue line can be drawn by clicking the “*Measure*” button. When the line is drawn, the pixel length of the blue line will appear in the “*Length (pixel)*” textbox. The user can type the corresponding length of the blue line in the “*Length (mm)*” textbox. Then, the application automatically converts the selected values into metric units.

To select the foreground (i.e., seed in this case), the user can click the “*foreground*” button, which will open a new window. The user can scribble on the seed using a red mark (see Figure 3A). In cases where the seed is too small, the user can zoom the image inward for scribbling. Thereafter, “*Original Image*” view can be restored. To select the background, the user can click the “*background*” button, which will open a new window. Then, the user can scribble on the background using a green mark (see Figure 3B).

Once the foreground and background have been marked or selected, the user can click the “*GraphCut*” button to segment the seeds from the background. An image showing the mask of the foreground will be shown in “*Processed Image*” view. After selecting the “*GraphCut*,” the histograms corresponding only to the seed region will be displayed in “*Channel 1*, *2*, and *3*” to guide the user in setting the color ranges. Implementation of the “*GraphCut*” function may take a few additional seconds. Supplementary Figure S5 shows the histogram and parameter setting with and without “*GraphCut*.”

Due to a wide range of variation in seed size and color, it is difficult to automatically set optimal size and color ranges (for all the color spaces). In this context, the tool provides the flexibility to the user to set these parameters manually based on the histograms. It is highly recommended that the user adjusts the parameters through testing. Nevertheless, the automatically generated default seed color and size parameters provide good initial values for the user to initiate adjusting parameters.

##### Scale Measurement

To obtain seed sizes in the metric system, the application allows the user to measure objects that have been used as a scale in the image (the tape in Figure 3C). The known size of the scale can be used to transform the pixel length into millimeters (mm), thus presenting the extracted trait values into the metric system. For this, a blue line can be drawn by clicking the “*Measure*” button. When the line is drawn, the pixel length of the blue line will appear in the “*Length (pixel)*” textbox. The user can type the corresponding length of the blue line in the “*Length (mm)*” textbox (see Figure 2). Then, the application automatically converts those values into metric units.

##### Testing and Processing

Once the user has set the parameters to investigate how the parameters work, a test should be performed prior to batch processing. To test the performance of the current parameters, the user can click the “*Test*” button. An image showing the mask of the seeds will be shown in “*Processed Image*” plot. There is a checkbox “*Seed Number*,” which is used to control whether the seed regions in the processed image will be numbered or not. If the box is ticked, a series of numbered yellow boxes will be drawn on the lower right corners of the individual seed in the binary image.

If the user has decided on the parameters to be used, batch processing can be initiated. The application requires that the seeds are not touching each other when imaged. The processing will begin by clicking the “*Launch*” button. A series of traits will be extracted by the application, and the extracted traits will be exported as CSV files. The “*Light bulb*” will turn red during the processing of images and will turn green upon completion of the designated task. The “*Progress*” gauge will show the progress of the image processing.

For each processed image, *SeedExtractor* will generate an output file that contains trait information of an individual seed in a particular image. Likewise, the mask of the seed regions from each image will be generated as a processed image. The indices of all the seed regions are marked in the processed image. In addition, the user can download combined file (labelled as TotalResult.csv) representing the average of particular trait for all the seeds per image from the MATLAB console.

### Algorithms

#### Image Segmentation

The foreground with the seeds needs to be segmented from the background to process the image. We use the color thresholding technique to find the seed regions. We allow the user to segment the images in one of the three color spaces, RGB, HSV, and Lab. The default color space is HSV, as we observed that HSV and Lab color spaces are better able to account for potentially uneven illuminations in the images. Range (minimum *C*_*i_ min*_ and maximum *C*_*i_ max*_) of the *i*th channel in the color parameter setting is used to define the color ranges in the selected color space. More specifically, if *C*_*1*_, *C*_*2*_, and *C*_*3*_ are the three values of a pixel in the selected color space, a pixel satisfying the following inequalities will be identified as a seed pixel:

$$C_{1\,\_\,m\,i\,n}<C_{1}<C_{1\,\_\,m\,a\,x}\&\&C_{2\,\_\,m\,i\,n}<C_{2}<C_{2\,\_\,m\,a\,x}\&\&C_{3\,\_\,m\,i\,n}<C_{3}<C_{3\,\_\,m\,a\,x}$$

where && means the logic *and* operation. The processed image that is used as the mask of seed regions can be generated after color thresholding in the selected color space (Bruce et al., 2000).

The application detects each seed region in the binary format. The shape-related traits are extracted from the binary or processed seed image and the colors are extracted from the original color image. Currently, this application provides a series of traits such as seed number, area, perimeter, length, width, circularity, and centroid, as well as seed-color intensity (see Table 1). In this software, area *A* is dictated by the number of pixels inside the region, and perimeter *P* is determined by the length of the boundary of the region. Major (seed length) and minor (seed width) axis lengths are the lengths of the major and the minor axis of the ellipse that has the same normalized second-central moments as the region. Circularity is calculated as (4π*A*)/*P*^2^ and can be used to evaluate how similar the region is to a circle. The centroid is the center of the seed region, which contains two values of coordinates. Color intensities are the average intensity of Red, Green, and Blue channel intensity values for each seed region.

**TABLE 1:** Traits evaluated by *SeedExtractor*.

Seed number
Area
Perimeter
Major axis length
Minor axis length
Circularity
Centroid
Color

#### Performance Testing

To test the performance of *SeedExtractor*, we evaluated the time required to process: (case-I) images having a different number of seeds and (case-II) images at different levels of resolution (see Supplementary Tables S1, S2 and Supplementary Figure S1). For this, mock seeds were computationally generated and increased from 1 seed to 100 seeds in a series of images (case-I). In case-II, we used a fixed number of 10 seeds, and increased the level of resolution of each image from 50 × 50 to 1,000 × 1,000 pixels.

#### Comparisons With Other Automated Methods and Manual Measurements

First, we compared the time taken by *SeedExtractor* to analyze images (10 mature seed images from different rice) compared to other freely available applications such as *SmartGrain* and *GrainScan* (see Supplementary Table S3). Next, we compared the accuracy of the seed morphometric measurements obtained by *SeedExtractor*, *SmartGrain*, and *GrainScan* to manual measurements using carbon fiber composite digital caliper (Resolution: 0.1 mm/0.01,” Accuracy: ± 0.2 mm/0.01,” Power: 1.5 V; Fisherbrand). For this, we only considered seed length as it can be manually measured with relatively higher confidence levels than seed width. Raw values from manual and image-based measurements are provided in Supplementary Table S4.

#### Seed Analyses From Other Plant Species

To show adaptability of the application to measure seed images from other plant species, we analyzed images scanned using flatbed scanner (controlled light conditions) from rice, wheat, soybean, sorghum, common bean, and sunflower (see Supplementary Figure S2). These plant species represent a wide variation in the seed size. Further, two additional users analyzed mature seed images from five different plant species to test the consistency of the *SeedExtractor*. The parameters used by the two users are presented in Supplementary Figure S3. In addition, to test the efficiency of our tool under variable light conditions and background, we analyzed developing rice seed (7 and 10 days after fertilization) images taken by a standard smartphone camera (12-megapixel, *f*/1.8 aperture; see Supplementary Figure S4).

### Rice Diversity Panel 1: A Test Case for *SeedExtractor* Validation

Approximately 231 rice accessions from RDP1 (Liakat Ali et al., 2011; Zhao et al., 2011; Eizenga et al., 2014) were grown under optimal greenhouse conditions, 16 h light and 8 h dark at 28 ± 1°C and 23 ± 1°C, respectively, and a relative humidity of 55–60% (Dhatt et al., 2019). The harvested panicles were dried (30 ± 1°C) for 2 weeks and mature seeds were dehusked using a Kett TR-250. The dehusked seeds were scanned using flatbed scanner—Epson Expression 12000 XL at 600 dpi resolution (Paul et al., 2020). The seeds were spread out on a transparent plastic sheet placed on the glass of the scanner to avoid scratching. A piece of tape at 0.5-inch (12.7 mm)width was used for scaling.

### Morphometric Measurements

*SeedExtractor* was used to obtain morphometric measurements on mature seed size. The various morphometric measurements derived from the scanned seed images were checked for normality and outliers were removed. The mature seed size data (length and width) was analyzed, and adjusted means for each accession across the replications were obtained with the following statistical model:

$${\text{y}}_{i\,k}=\mu +{\text{g}}_{i}+{\text{r}}_{k}+\epsilon_{i\,k}$$

where **y**_*ik*_ refers to the performance of the *i*th accession in the *k*th replication, μ is the intercept, **g**_*i*_ is the effect of the *i*th accession, **r**_*k*_ is the effect of *k*th replication, and ϵ_*ik*_ is the residual error associated with the observation **y**_*ik*_. R statistical environment was used for the analysis (R Core Team, 2019).

### Genome Wide Association Study (GWAS)

Adjusted means of various seed morphometric were used for GWAS analysis. GWAS was performed in rrBLUP R package (Endelman, 2011) using a high-density rice array (HDRA) of a 700k single nucleotide polymorphism (SNP) marker dataset (McCouch et al., 2016) with a total of 411,066 SNPs high quality SNPs retained after filtering out the missing data (<20%) and minor allele frequency (<5%). Following single marker linear mixed model was used for GWAS:

y=1⁢μ+X⁢β+s⁢α+Z⁢g+ϵ

where **y** is a vector of observations, μ is the overall mean, **X** is the design matrix for fixed effects, β is a vector of principle components accounting for population structure, **s** is a vector reflecting the number of alleles (0,2) of each genotype at particular SNP locus, α is the effect of the SNP, **Z** is the design matrix for random effects, $\text{g}∼N\,\left(0,\text{G}\,\sigma_{g}^{2}\right)$ is the vector of random effects accounting for relatedness, **G** is the genomic relationship matrix of the genotypes, $\sigma_{g}^{2}$ is the genetic variance, and ϵ is the vector of residuals. Manhattan plots were plotted using the qqman R package (Turner, 2014). To declare the genome-wide significance of SNP markers, we used a threshold level of *P* < 3.3 × 10^–6^ *p* or -log_10_(*P*) > 5.4 (Bai et al., 2016).

## Results and Discussion

### Performance Test

**FIGURE 4:**
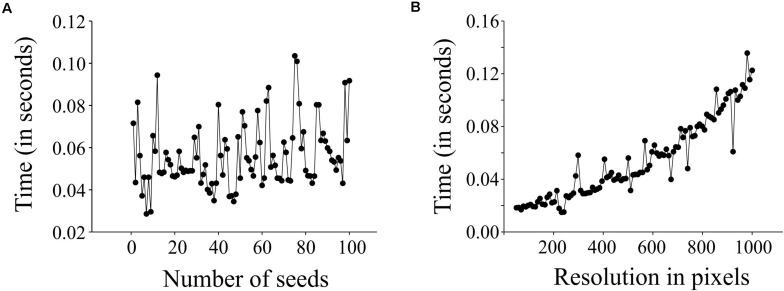
Performance testing of *SeedExtractor*. Plot showing the time taken to process images having different number of seeds **(A)**, and images having different resolution levels **(B)**.

We evaluated the performance of *SeedExtractor* with respect to the time required to process images. For this, we evaluated two cases: images having different numbers of seeds and images at different levels of resolution. In the first case, we used an incremental range (from 1 to 100) of seeds in a series of images (see Supplementary Table S1 and Supplementary Figure S1A). We observed that the number of seeds does not affect the performance, as the time taken to process an image with a single seed is similar to that of an image with 100 seeds (see Figure 4A). Secondly, we used a fixed number of seeds and increased the resolution of each consecutive image incrementally (see Supplementary Table S2 and Supplementary Figure S1B). We detected that the performance of the application slows gradually with increase in resolution (see Figure 4B).

### *SeedExtractor* vs. Other Automated Software and Manual Measurements

Next, we investigated the efficiency of *SeedExtractor* with respect to the time needed to analyze images relative to other automated software tools such as *SmartGrain* and *GrainScan*. Remarkably, the *SeedExtractor* takes ∼21 s for analyzing 10 images i.e., 30 times and 6 times more efficient than *SmartGrain* and *GrainScan*, respectively (see Supplementary Table S3). Then, we correlated manual measurements with the analysis performed using each of the three automated software (*SeedExtractor, SmartGrain*, and *GrainScan*). Although manual measurements are prone to errors, we considered only seed length for the correlation because it can be measured with relatively higher confidence levels than seed width. Consequently, *SeedExtractor* showed the least deviation from manual measurements, as we detected correlation of 0.93 for *SeedExtractor*, 0.84 for *GrainScan*, and 0.92 for *SmartGrain* with manually measured seed length (see Supplementary Table S4). Furthermore, we checked the correlation between the morphometric measurements obtained from the *SeedExtractor* and the other two software (see Table 2 and Supplementary Table S5). We detected a significantly high correlation (>0.97) between the analyses conducted by *SeedExtractor* and *SmartGrain* (see Table 2 and Supplementary Table S5). Contrarily, the correlation between *GrainScan* and *SmartGrain* or *SeedExtractor* was relatively low (<0.81; see Table 2 and Supplementary Table S5). Thus, *SeedExtractor* serves in a time-efficient and reliable manner to analyze seed size parameters.

**TABLE 2:** Correlation of the three automated applications for determining different seed size parameters.

Trait	*GrainScan and*	*GrainScan and*	*SmartGrain and*
	*SmartGrain*	*SeedExtractor*	*SeedExtractor*
Area	0.273	0.229	0.986
Perimeter	0.313	0.346	0.979
Length	0.549	0.518	0.995
Width	0.814	0.808	0.994
Axis Ratio	NA	NA	0.998
Circularity	NA	NA	0.923

**For comparisons, mature seeds from one rice accession was considered. NA, not applicable.**

### Seed Image Analysis From Other Species

**FIGURE 5:**
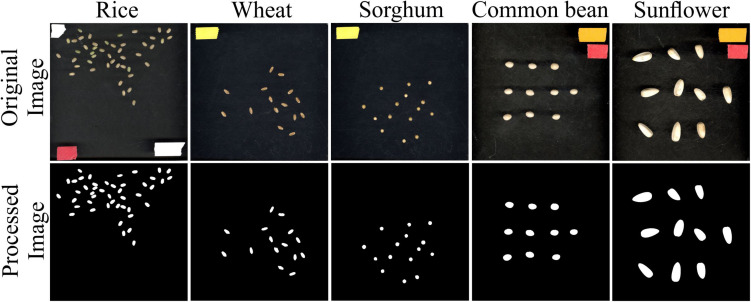
Seed analysis of different plant species. Mature seed images (original image) corresponding to rice, wheat, sorghum, common bean, and sunflower were evaluated using *SeedExtractor*. Processed image shows the segmented image pertaining to their respective plant species. Different color tapes in the original image were used for scaling purposes.

In addition to rice, seed measurements from other plant species representing a wide variation with respect to seed size were evaluated using *SeedExtractor*. For this, mature seeds from wheat, sorghum, common bean, and sunflower were also processed using *SeedExtractor*. After establishing the optimal parameters (see Supplementary Figure S2 and Supplementary Table S6), *SeedExtractor* precisely segmented the mature seeds form the different plant species (see Figure 5). Further, these values were consistent with those obtained from two additional independent users analyzing these images (see Supplementary Figure S3 and Supplementary Tables S6, S7, S8).

Since the analyzed seed images were taken in controlled light conditions (flatbed scanner Epson Expression 12000 XL), we determined the efficacy of the tool by analyzing developing rice seed images taken by a standard smartphone camera under variable light conditions and background. As a result, we were able to finely segment the developing rice seeds from the background using *SeedExtractor* (see Supplementary Figure S4). In summary, the successful and consistent derivation of the seed morphometrics from multiple plant species as well as the ability to analyze seed images taken under controlled and variable light conditions demonstrates the adaptability and utility of the application.

### Validation of *SeedExtractor* Derived Morphometric and Colorimetric Data

**FIGURE 6:**
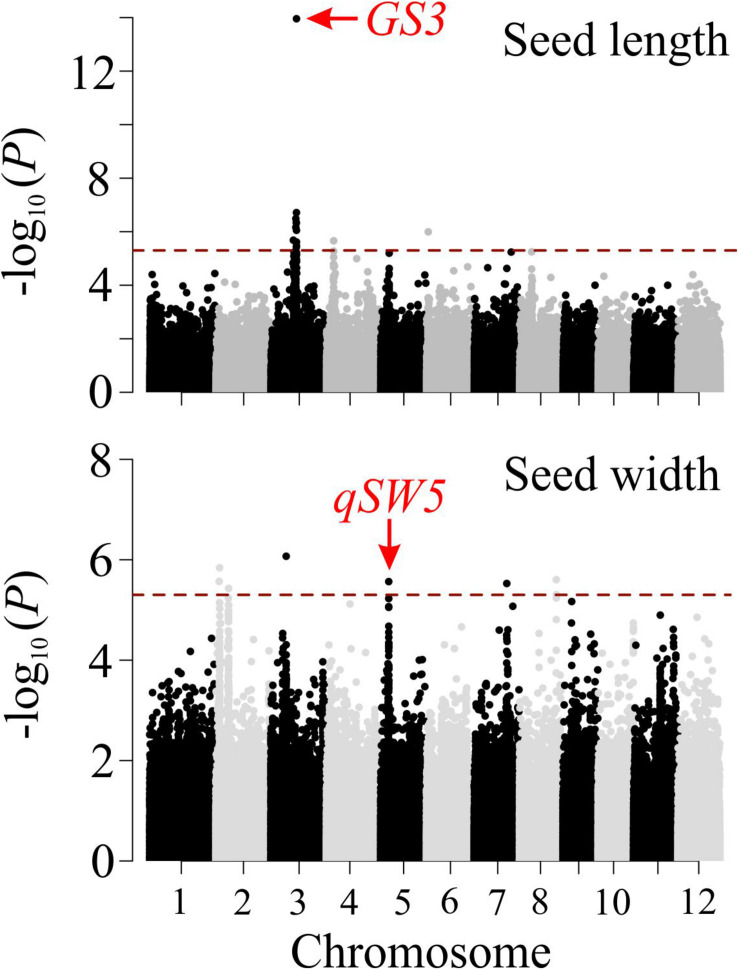
Manhattan plots of genome-wide association analysis for mature grain length (upper panel) and width (lower panel). The red dashed horizontal line indicates cut-off of significance threshold (*P* < 3.3 × 10^– 6^ or -log_10_(*p*) > 5.4) level. Previously known major seed length (*GS3*) and width (*qSW5*) regulators are highlighted with a red arrow.

To validate the seed related traits derived from *SeedExtractor*, we screened 231 rice accessions corresponding to RDP1 (see Supplementary Table S9). The mature seed length and width, which showed a normal distribution, were used for GWAS (see Supplementary Figure S6). Consequently, we identified 13 significant SNPs associated with seed length and 8 with seed width under control (see Figure 6 and Supplementary Table S10). Remarkably, the lead SNP on chromosome 3 (SNP3.16732086; -log_10_ *P* = 13.95) that affects mature seed length, corresponded to *GS3*, a known regulator of seed size (Fan et al., 2006). This known regulation of *GS3* was explanatory for 13.24% of phenotypic variation (Figure 6 and Supplementary Table S8). *GS3* encodes a subunit of G-protein complex. Different alleles of *GS3* have been discussed to promote either longer (null alleles; Fan et al., 2006; Takano-Kai et al., 2009) or shorter seeds (gain-of-function allele; Mao et al., 2010). The other two significant SNPs for grain length were detected on chromosome 4 (SNP4.4655556; -log_10_ *P* = 5.66) and 6 (SNP6.1112028; -log_10_ *P* = 5.99), which encompasses *deformed interior floral organ 1* and an expressed protein, respectively.

Furthermore, we identified several SNPs for seed width (see Supplementary Table S10). For instance, the lead SNP on chromosome 2 (SNP2.2487459; -log_10_ *P* = 6.07) co-localizes with an expressed protein (*Os02g05199*), and the SNP on chromosome 3 (SNP3.10130641; -log_10_ *P* = 5.84) is localized in the intergenic sequence between *Os03g18130* and *Os03g18140* (see Figure 6 and Supplementary Table S10). Interestingly, the significant SNP on chromosome 5 (SNP5.5348012; -log_10_ *P* = 5.56; see Figure 6 and Supplementary Table S10) corresponded to a known regulator for seed width, *qSW5/GW5* (Weng et al., 2008; Duan et al., 2017; Liu et al., 2017; Kumar et al., 2019). This SNP explained phenotypic variation of 4.4%, which is in line with the previous studies (Huang et al., 2010; Zhao et al., 2011). The detection of the known seed size regulators, and the novel loci from the association mapping of the morphometric data, obtained by *SeedExtractor*, substantiates the power of the application to facilitate trait discovery.

Next, to test *SeedExtractor’s* capability to extract colorimetric features, we screened the RDP1 that have already been visually classified based on seed color (Liakat Ali et al., 2011; Zhao et al., 2011; Eizenga et al., 2014). We detected a clear distinction between the *SeedExtractor* derived color intensities that corresponded to different color-based groups (see Supplementary Figure S7 and Supplementary Table S11). Collectively, these results validate the robustness of *SeedExtractor’s* ability to analyze seed size, shape, and color parameters that can be used in downstream genetic analysis for trait discovery.

## Conclusion

This open-source cross-platform application provides a powerful tool to analyze seed images from a wide variety of plant species in a time-efficient manner. The accuracy of the tool is demonstrated by GWAS that identified the known regulators of seed length and width in rice. The versatility of this tool can extend beyond flatbed-scanned images, as it can also evaluate images taken by other cameras. In the future, this tool can be extended to include downstream processing of the results (e.g., phenotypic distribution and clustering) as well as to estimate other yield-related parameters such as opaqueness or chalkiness in rice, which account for significant yield losses in global rice production.

## Data Availability Statement

All datasets generated for this study are included in the article/Supplementary Material, further inquiries can be directed to the corresponding author/s.

## Author Contributions

HW and HY supervised the project. PP led the study, scanned the seeds, and performed manual measurements. PP, BD, JS, LI, and KW performed the experiment on Rice Diversity Panel 1. FZ designed and developed the application. WH and GM performed analysis on the phenotypic data and genome-wide association mapping. PP and HW performed candidate gene analysis. PP and FZ wrote the manuscript. All authors read and approved the manuscript.

## Conflict of Interest

The authors declare that the research was conducted in the absence of any commercial or financial relationships that could be construed as a potential conflict of interest.

## Supplementary Material

Supplementary Figure 1 Images used for performance testing.

Supplementary Figure 2 Parameters used to evaluate images from multiple plant species.

Supplementary Figure 3 Seed color and size parameters used by User-1 and User-2.

Supplementary Figure 4 Rice developing seed at 7 and 10 days after fertilization analyzed using *SeedExtractor*.

Supplementary Figure 5 Graph-cutting.

Supplementary Figure 6 Phenotypic distribution of mature seed length and width.

Supplementary Figure 7 Box plot representing seed color intensities for three channels in RGB color space for the visually classified RDP1. The numbers below the color groups signify the number of genotypes in the respective group. For stats, we used LSmeans student’s *t*-test across all 15 groups (5 color groups and 3 color channels). Different letters indicate significant differences between a particular group and channel; α = 0.05 and t-statistic = 1.96.

Supplementary Table 1 Performance testing with incremental seed numbers.

Supplementary Table 2 Performance testing with incremental image resolution.

Supplementary Table 3 Evaluation of efficiency with respect to time.

Supplementary Table 4 Manual and automated seed length measurements.

Supplementary Table 5 Morphometric analysis using the automated applications.

Supplementary Table 6 *SeedExtractor* based analysis of seed images from different plants.

Supplementary Table 7 Morphometric measurements of mature seed images from different plant species using *SeedExtractor* by two additional users (User-1 and User-2). Color and size parameters by User-1 and 2 are also represented.

Supplementary Table 8 Variation in (area and color-1) of mature seed images from different plant species using *SeedExtractor* by two additional users (User-1 and User-2).

Supplementary Table 9 Rice accessions used for genome wide association study.

Supplementary Table 10 Significant SNPs associated with seed length and width.

Supplementary Table 11 Seed color intensities for three channels in RGB color space for the RDP1.

Supplementary Data Sheet 1 *SeedExtractor* guide document.
