# Supplementary figures and images for: SeedExtractor: An Open-Source GUI for Seed Image Analysis
Source: Front Plant Sci. 2021 Feb 1;11:581546. doi: 10.3389/fpls.2020.581546 (PMC7882627; doi:10.3389/fpls.2020.581546)

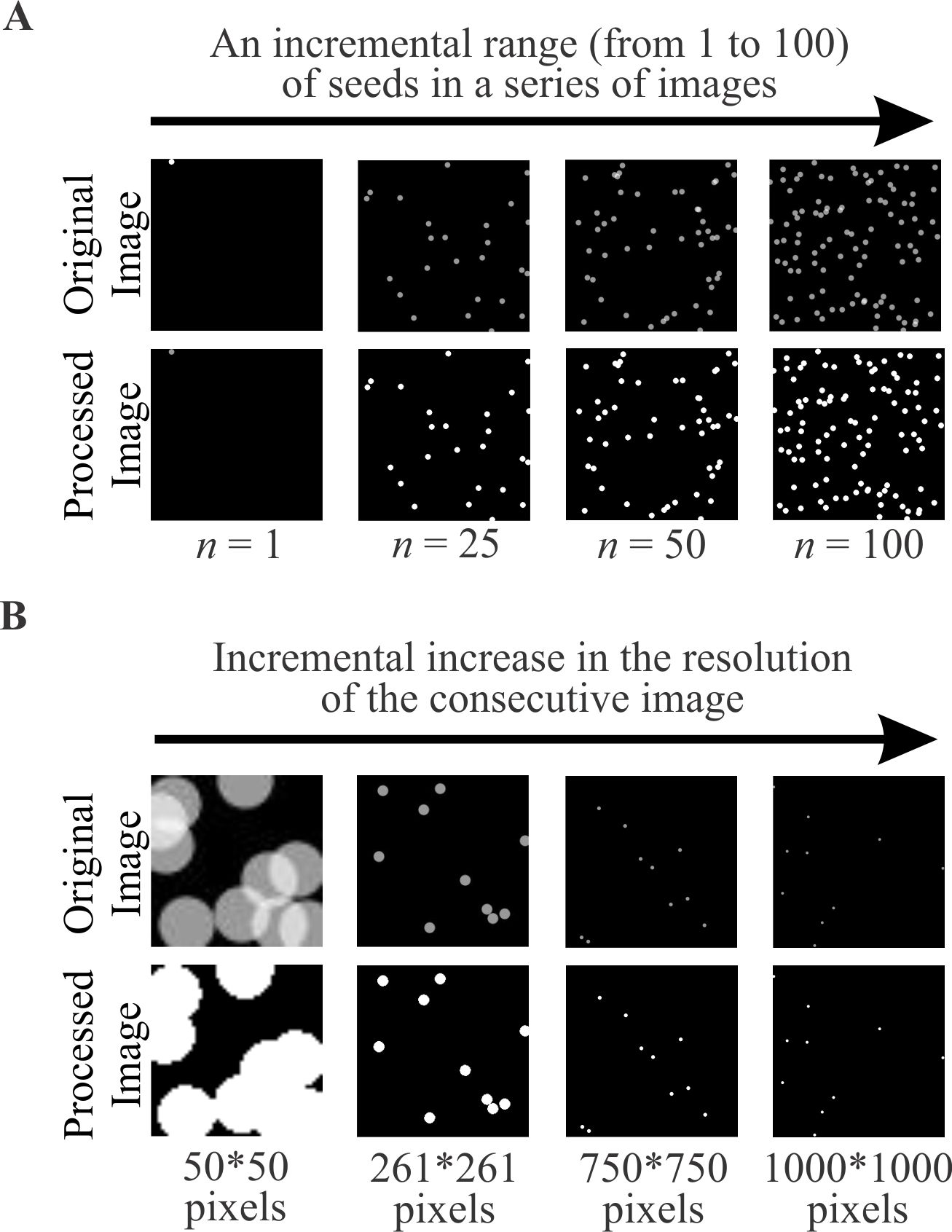

Supplement: Supplementary Figure 1 — Images used for performance testing. [file Image_1.JPEG]

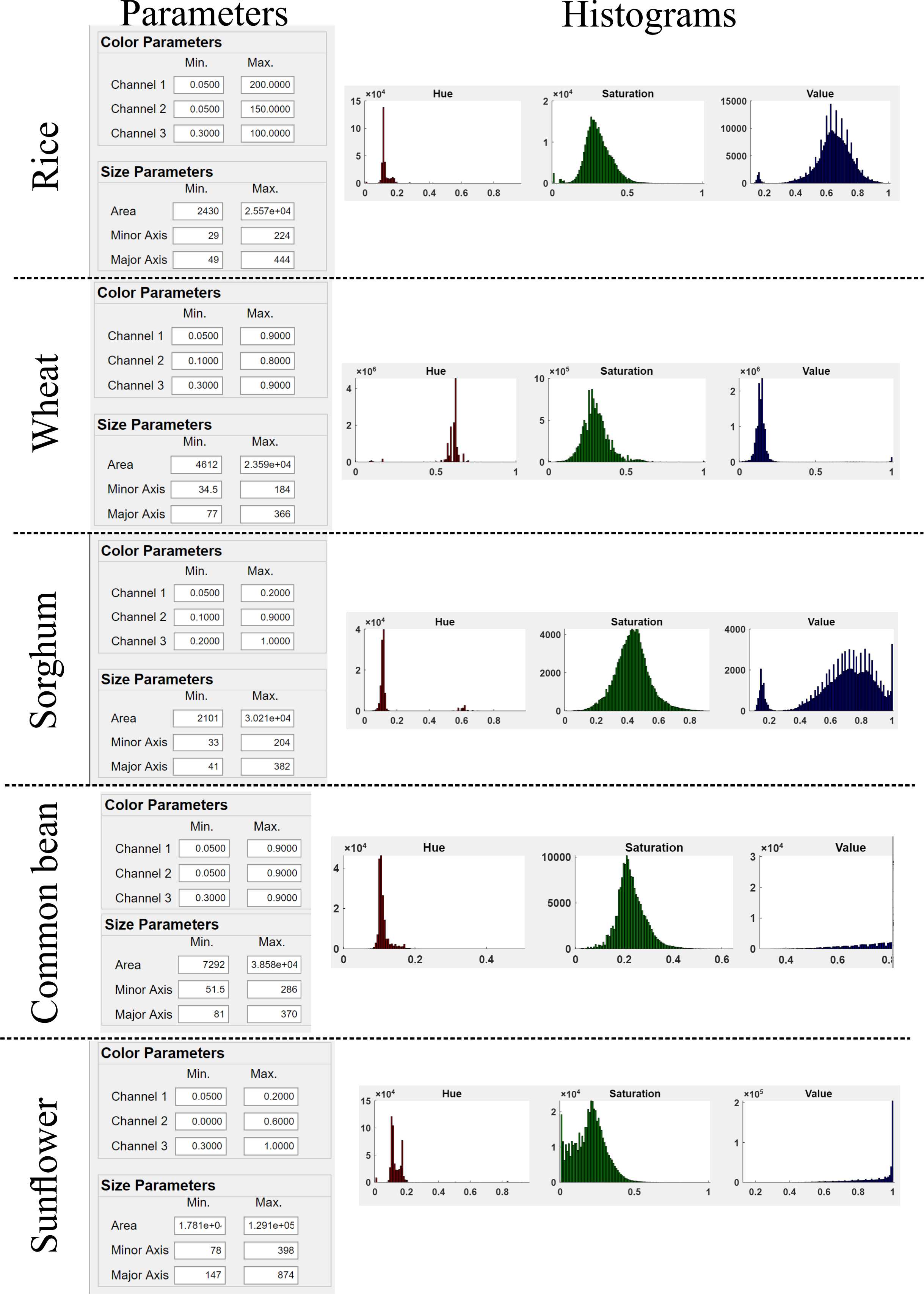

Supplement: Supplementary Figure 2 — Parameters used to evaluate images from multiple plant species. [file Image_2.JPEG]

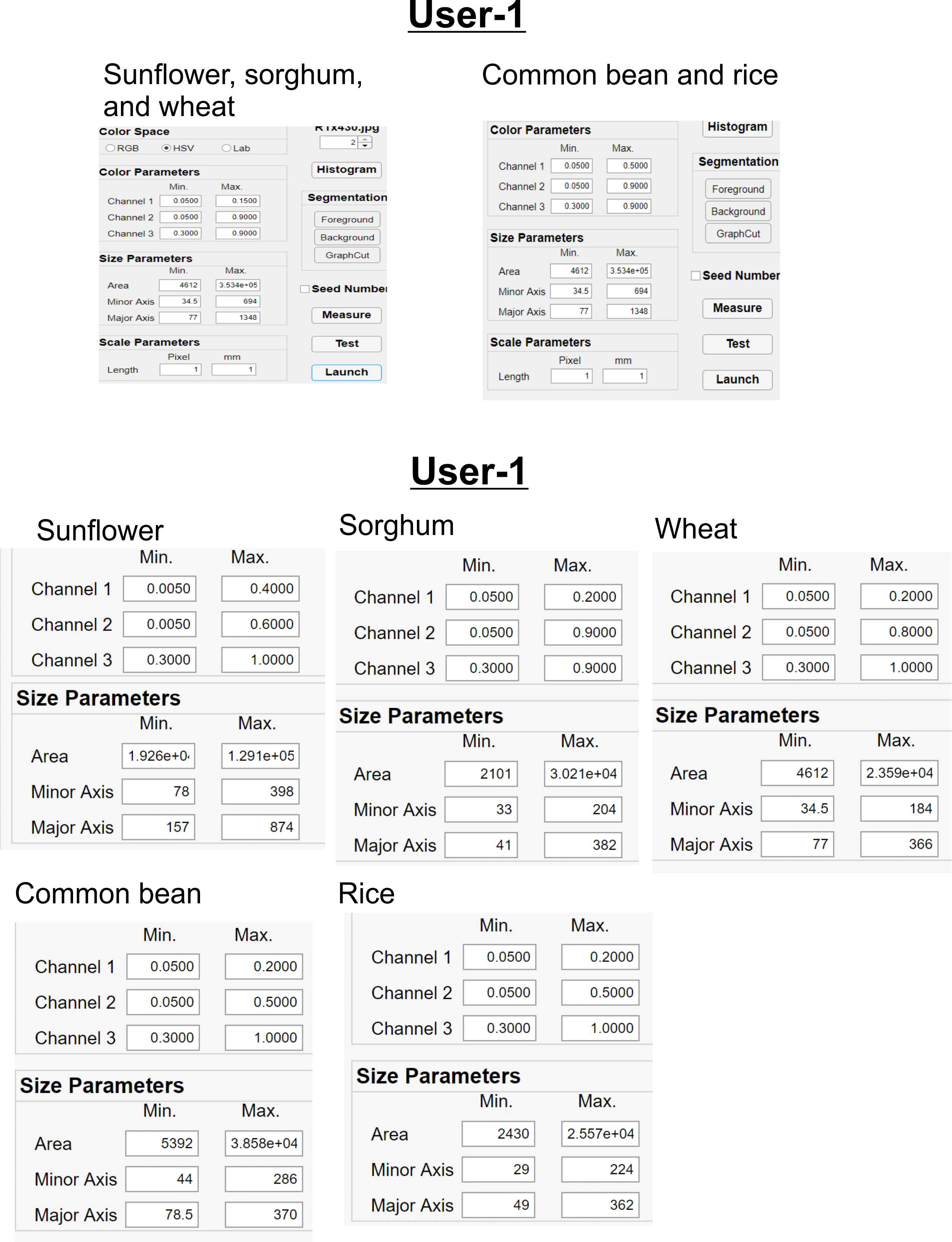

Supplement: Supplementary Figure 3 — Seed color and size parameters used by User-1 and User-2. [file Image_3.JPEG]

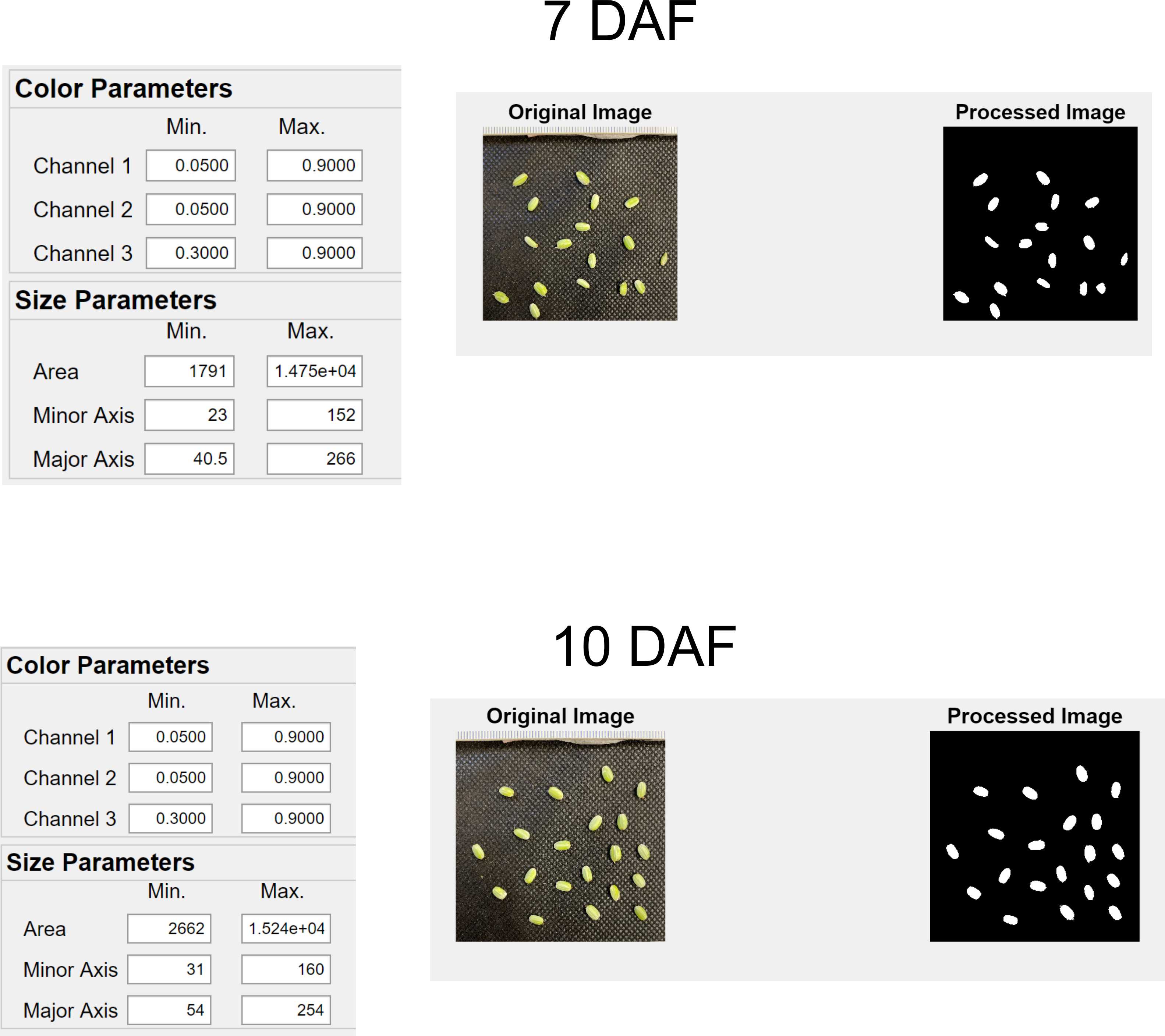

Supplement: Supplementary Figure 4 — Rice developing seed at 7 and 10 days after fertilization analyzed using SeedExtractor. [file Image_4.JPEG]

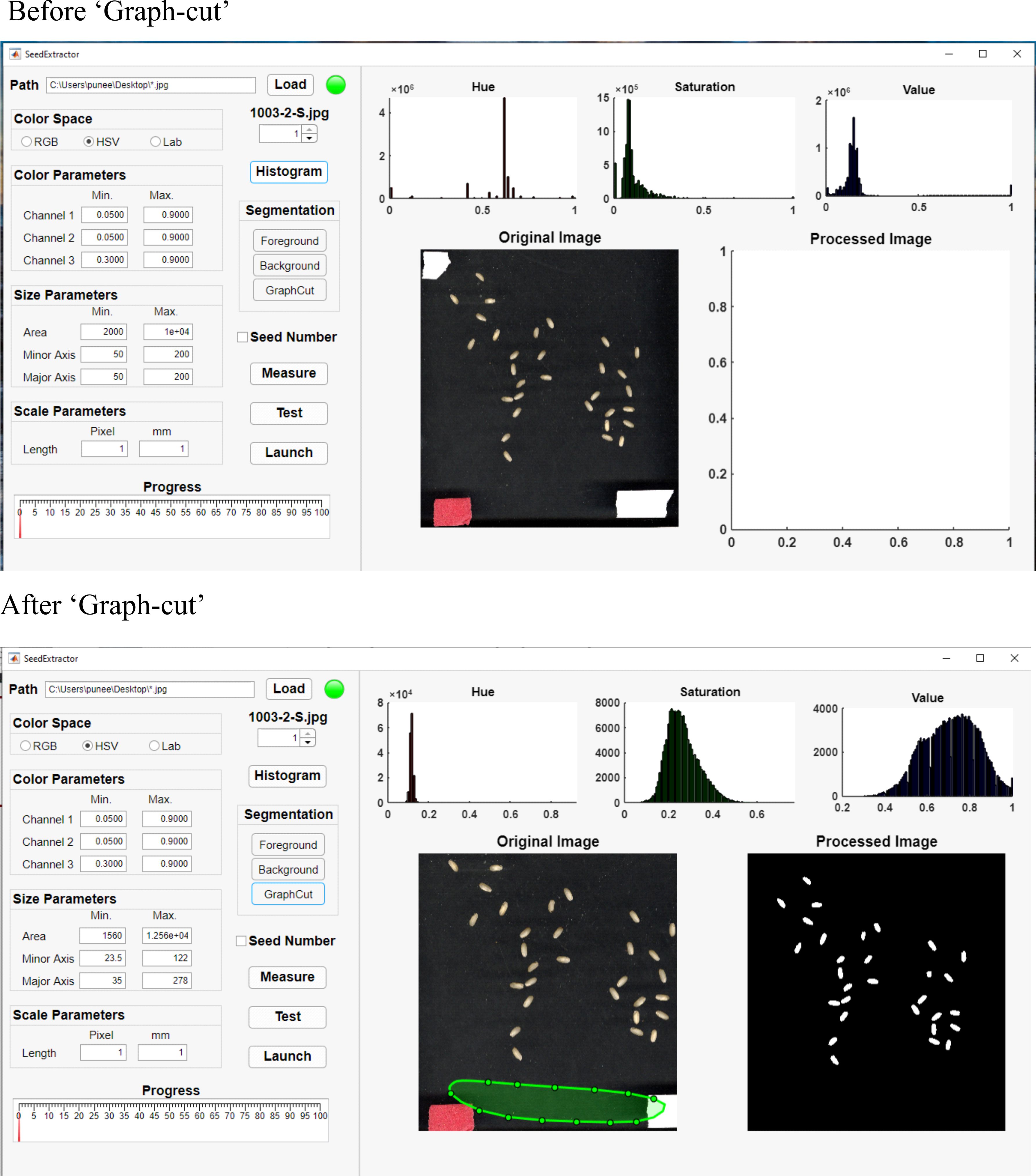

Supplement: Supplementary Figure 5 — Graph-cutting. [file Image_5.JPEG]

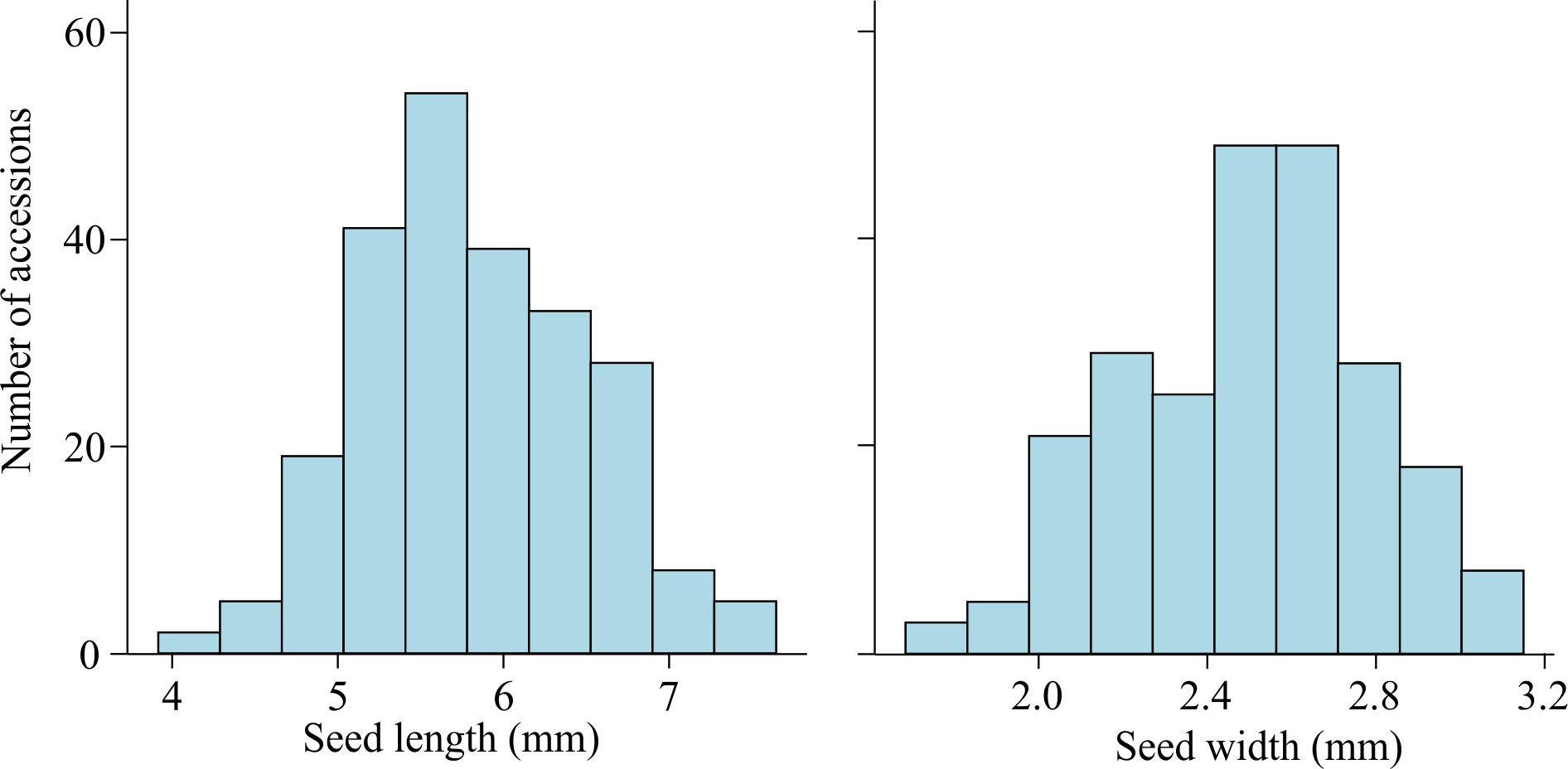

Supplement: Supplementary Figure 6 — Phenotypic distribution of mature seed length and width. [file Image_6.JPEG]

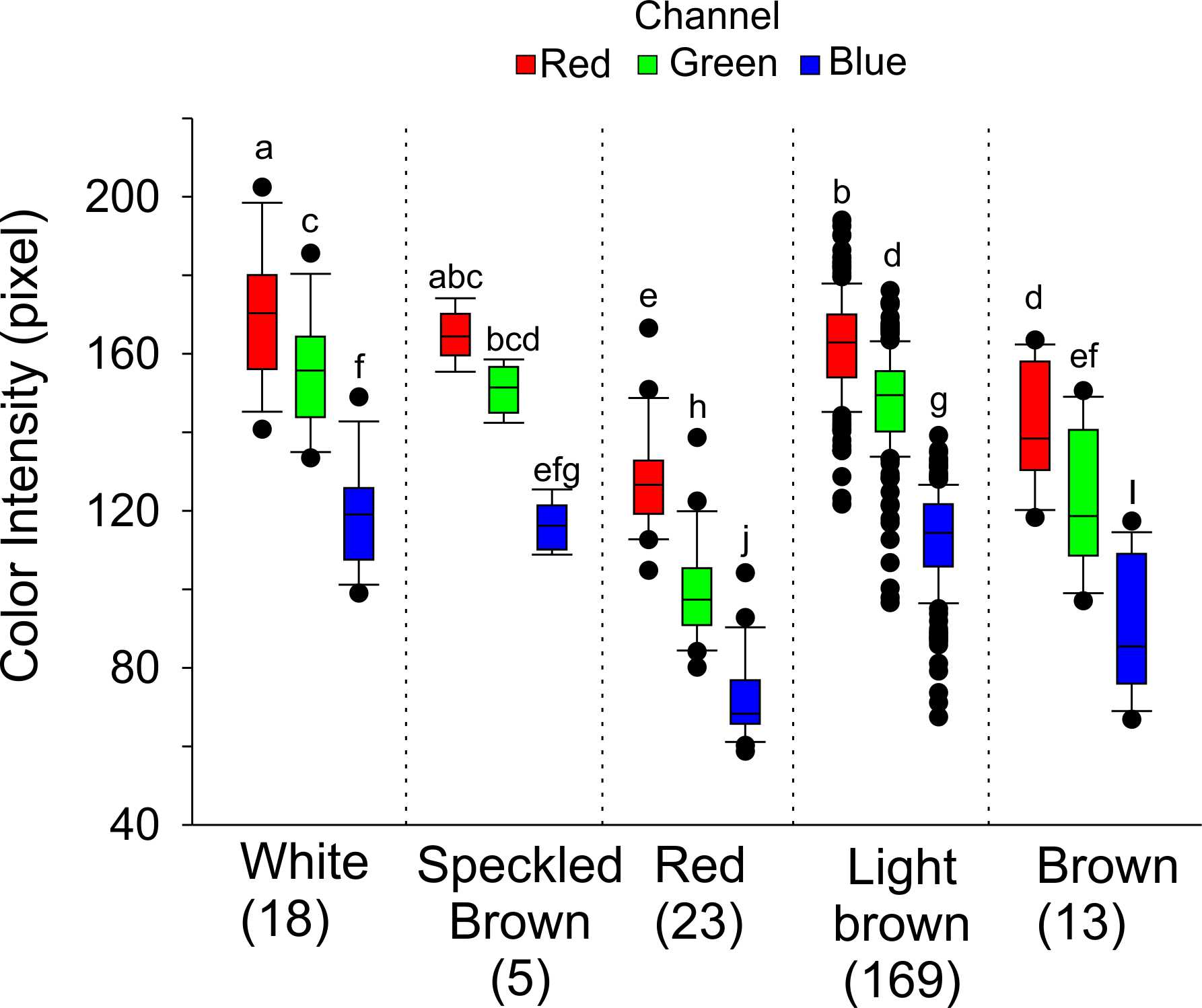

Supplement: Supplementary Figure 7 — Box plot representing seed color intensities for three channels in RGB color space for the visually classified RDP1. The numbers below the color groups signify the number of genotypes in the respective group. For stats, we used LSmeans student’s t-test across all 15 groups (5 color groups and 3 color channels). Different letters indicate significant differences between a particular group and channel; α = 0.05 and t-statistic = 1.96. [file Image_7.JPEG]
